# Aminoguanidine Prevents the Oxidative Stress, Inhibiting Elements of Inflammation, Endothelial Activation, Mesenchymal Markers, and Confers a Renoprotective Effect in Renal Ischemia and Reperfusion Injury

**DOI:** 10.3390/antiox10111724

**Published:** 2021-10-28

**Authors:** Consuelo Pasten, Mauricio Lozano, Jocelyn Rocco, Flavio Carrión, Cristobal Alvarado, Jéssica Liberona, Luis Michea, Carlos E. Irarrázabal

**Affiliations:** 1 Laboratorio de Fisiología Integrativa y Molecular, Programa de Fisiología, Centro de Investigación e Innovación Biomédica, Universidad de los Andes, Santiago 7620157, Chile; mpasten@uandes.cl (C.P.); mlozano@uandes.cl (M.L.); joce.valeska.roccog@gmail.com (J.R.); 2 Facultad de Medicina, Universidad de los Andes, Santiago 7620157, Chile; 3 Facultad de Ciencias de la Salud, Universidad del Alba, Santiago 7620157, Chile; flavio.carrion@udalba.cl; 4 Clinical Research Unit, Hospital Las Higueras, Talcahuano 4260000, Chile; cristobalalvarado@ucsc.cl; 5 Department of Basic Sciences, School of Medicine, Universidad Católica de la Santísima Concepción, Concepción 4030000, Chile; 6 Instituto de Ciencias Biomédicas, School of Medicine, Universidad de Chile, Santiago 7620157, Chile; jliberona@clinicauandes.cl (J.L.); lmichea@med.uchile.cl (L.M.); 7 Millennium Institute on Immunology and Immunotheraphy, Santiago 762015, Chile

**Keywords:** aminoguanidine, antioxidants, oxidative stress, ischemia-reperfusion injury, renal protection

## Abstract

Oxidative stress produces macromolecules dysfunction and cellular damage. Renal ischemia-reperfusion injury (IRI) induces oxidative stress, inflammation, epithelium and endothelium damage, and cessation of renal function. The IRI is an inevitable process during kidney transplantation. Preliminary studies suggest that aminoguanidine (AG) is an antioxidant compound. In this study, we investigated the antioxidant effects of AG (50 mg/kg, intraperitoneal) and its association with molecular pathways activated by IRI (30 min/48 h) in the kidney. The antioxidant effect of AG was studied measuring GSSH/GSSG ratio, GST activity, lipoperoxidation, iNOS, and Hsp27 levels. In addition, we examined the effect of AG on elements associated with cell survival, inflammation, endothelium, and mesenchymal transition during IRI. AG prevented lipid peroxidation, increased GSH levels, and recovered the GST activity impaired by IRI. AG was associated with inhibition of iNOS, Hsp27, endothelial activation (VE-cadherin, PECAM), mesenchymal markers (vimentin, fascin, and HSP47), and inflammation (IL-1β, IL-6, Foxp3, and IL-10) upregulation. In addition, AG reduced kidney injury (NGAL, clusterin, Arg-2, and TFG-β1) and improved kidney function (glomerular filtration rate) during IRI. In conclusion, we found new evidence of the antioxidant properties of AG as a renoprotective compound during IRI. Therefore, AG is a promising compound to treat the deleterious effect of renal IRI.

## 1. Introduction

Aminoguanidine (AG) is a small, nontoxic molecule with different biological actions. It has been shown that AG inhibits the formation of highly reactive advanced glycosylation end products (AGEs) associated with diabetes mellitus (DM) and decreases the complications related to proteinuria [1], retinopathy [2], and neuropathy [3]. Due to these effects, AG was proposed as a therapeutic agent inhibiting AGEs formation [4]. However, in a phase III clinical trial in type 1 diabetic patients, aminoguanidine reduced proteinuria and retinopathy, whereas the progression to overt nephropathy was not statistically improved. In addition, a high dose of aminoguanidine provoked abnormalities in liver function and other side effects associated with flu-like symptoms, gastrointestinal alterations, rare vasculitis, and anemia [5]. However, the beneficial therapeutic properties of low doses of AG in other kidney diseases are poorly understood.

In another view, AG is a potent antioxidant compound and free radical scavenger. High doses (AG ≥ 5 mM) have an inhibitory effect on the xanthine oxidase (XO) activity [6]. AG also is an effective in vitro quencher of reactive carbonyl species (RCS) [7]. The radical scavenging properties of AG may contribute to protective effects during glycation and explain the prevention of diabetic complications. In a model of streptozotocin-induced diabetes, AG demonstrated antioxidant effects decreasing plasma lipid hydroperoxide and thiobarbituric acid (TBARS) [8]. AG also showed antioxidant action against brain injury induced by doxorubicin (DOX), decreasing the levels of MDA (malondialdehyde) and GPx (Glutathione peroxidase) and increasing the GST (glutathione S-transferase) activity [9]. Similarly, in a model of intestinal IRI, administration of AG reduced the intestinal mucosal injury and decreased MDA, protein carbonyl (PC), SOD (superoxide dismutase), and GPx levels [10].

Renal IRI induces hypoxia, oxidative stress, vasoconstriction, destruction of the tubular cells, endothelial cell damage, inflammation, cell necrosis, and cessation of renal function [11]. The reperfusion phase results in more production of reactive oxygen species (ROS) [12,13,14], including, among others, superoxide anion (O_2_^−^), hydrogen peroxide (H_2_O_2_), and hydroxyl radicals (OH·), all of which have chemical properties that confer reactivity to different biological targets [15,16]. Further, IRI injury led to a significant increase in NO synthesis, and high concentrations produce peroxynitrite (ONOO-), which interacts with lipids, DNA, and proteins, leading to cell damage [17]. We previously described a murine model of IRI and observed increased levels of oxidative stress and kidney disfunction (glomerular filtration rate, neutrophil gelatinase-associated lipocalin, and clusterin) [18]. Previously, the literature described that co-treatment with α-tocopherol and aminoguanidine during unilateral IRI (I/R: 2 h/24 h) prevented the formation of MDA and nitrite/nitrate in plasma [19]. Besides, in a bilateral renal IRI model (I/R 60 min/24 h), AG (100 mg /kg, i.p.) decreased the MDA levels but did not attenuate the histological damage [20], indicating an antioxidant effect of AG during renal IR. In addition, the posttreatment with AG (50 mg/kg, i.p.) in a model of bilateral ischemia (IR 60 min/24 h) significantly reduced serum urea and creatinine levels and improved histopathological lesions observed by IRI (45 min/24 h) [21]. Therefore, the role of AG during renal IRI is not very clearly understood.

Interestingly, AG has been described as an effective inhibitor of NOS, specifically the inducible NO synthase isoform (iNOS). AG suppresses the NO production after the reperfusion phase in experimental models of cerebral [22] or myocardial [23] IRI. AG suppresses iNOS activity in mice with brain ischemia to a level equivalent to those seen in iNOS knockout mice [24]. In the kidney, there are three different isoforms of nitric oxide synthase (NOS)—neuronal NOS (nNOS), endothelial NOS (eNOS), and inducible NOS (iNOS). While nNOS and eNOS are constitutively expressed, iNOS is a calcium-independent synthase whose expression is induced by cytokines, oxidative stress, and transcription factors such as NF-Ƙb [25]. We previously described that the pharmacological inhibition of iNOS inhibition (l-NIL) prevented oxidative stress and improved renal function during IRI [18]. On the other hand, the Hsp27 is a stress protein that shows an early and transient increase after acute ischemia [26], inhibiting apoptosis by decreasing intracellular reactive oxygen species and the mitochondrial caspase-dependent apoptotic pathway [27]. Selective renal overexpression of Hsp27 in mice through lentiviral gene delivery protects against ischemic renal injury [28]. Arginase-2 (Arg-2) and transforming growth factor-beta 1 (TGF-β1) are two markers of kidney damage by renal IR. Arg2 plays a significant role in renal fibrosis via its action on NO and mitochondrial function [29], and TGF-β is recognized as a central mediator of renal tubulointerstitial fibrosis [30]. Finally, new evidence suggests that the development of vascular rarefaction and fibrosis after an injury is dependent on the differentiation of renal endothelial and tubular cells into mesenchymal cells by IRI. These processes are called epithelial-to-mesenchymal transition (EMT) and endothelial-to-mesenchymal transition (EndMT), respectively [31,32]. EMT is reversible and leads to the loss of cellular polarity, migratory capacity development, resistance to apoptosis, and the induction of extracellular matrix synthesis [33]. EndMT resembles and leads to loss of cellular adhesion molecules, cytoskeletal reorganization, and change of the compacted cobblestone-like into spindle-shaped phenotype without polarity, associated with reduced expression of endothelial markers (e.g., vascular endothelial-cadherin, CD31/PECAM-1, and von Willebrand factor) [34]. Both EMT and EndMT show increased mesenchymal cell markers such as vimentin, fascin1, and Hsp47 [35,36].

This study aimed to investigate the antioxidant property of AG in the kidney and its potential renoprotective effect during renal IRI. We studied the effect of intraperitoneal injection of AG (50 mg/kg) before the IRI on reduced and oxidized glutathione ratio (GSH/GSSG), glutathione S-transferase (GST) activity, lipoperoxidation, iNOS, and Hsp27. In addition, we identified new molecular pathways of the antioxidant effect of AG implicated in inflammation, cell injury, endothelial activation, and mesenchymal transition to provide novel evidence of the renoprotective effect of AG during renal IRI.

## 2. Materials and Methods

### 2.1. Animals

Previously, we described an IRI model using male BALB/c mice [18]. The mice (20–25 g) were housed in a 12 h light/dark cycle. Animals were maintained at the University de los Andes Animal Care Facility with food and water ad libitum. All experimental procedures followed institutional and international standards for humane care and use of laboratory animals (Animal Welfare Assurance Publication A5427-01, Office for Protection from Research Risks, Division of Animal Welfare. The National Institutes of Health). All procedures were approved by the Committee on the Ethics of Animal Experiments of the University de los Andes, Chile.

### 2.2. Ischemia-Reperfusion (IR) Experiments

The animals were anesthetized with 25 mg/kg i.p. ketamine/15 mg/kg i.p. xylazine (Drag Pharma, Santiago, Chile) and maintained on a 37 °C blanket during the surgical procedure. A flank incision exposed both kidneys, and the renal pedicle was occluded for 30 min with a non-traumatic vascular clamp (cat N° 18055-02 Fine Science Tools). Renal blood flow was re-established (reperfusion phase) by clamp removal, and both incisions were sutured. Sham animals did not undergo renal pedicle occlusion [18,37]. To study the effects of AG, mice were treated intraperitoneally (i.p.) with either vehicle or 50 mg/kg of aminoguanidine (aminoguanidine hydrochloride, TOCRIS TO.0787/100) [21] before ischemia. Then, the animals were subjected to 48 h of reperfusion according to our previous publication [18]. Following protocols, mice were euthanized with CO_2_, and kidneys were dissected and processed for analysis.

### 2.3. Oxidative Stress Experiments

Kidney samples were homogenized in a Dounce homogenizer in 0.15 M potassium chloride (KCl). After measuring the total protein concentration [38], aliquots were deproteinized with cold trichloroacetic acid 30% *v*/*v* for 10 min, and the pellet was removed by centrifugation. Oxidative stress was evaluated in kidney homogenates as changes of the reduced glutathione (GSH) pool assayed in deproteinized kidney homogenates and lipid peroxidation, measured with the thiobarbituric acid reactive substances (TBARS) assays [39]. In addition, total enzymatic glutathione S-transferase (GST) activity was assayed in homogenates using 1-chloro-2,4-dinitrobenzene (CDNB) as a substrate and GSH as a cofactor [40].

### 2.4. Quantification of Neutrophil Gelatinase-Associated Lipocalin

Neutrophil gelatinase-associated lipocalin (NGAL) was quantified in plasma samples using a commercial ELISA kit (Bioporto) according to the manufacturer’s instructions.

### 2.5. Glomerular Filtration Rate (GFR) Measurements

GFR was determined in conscious unrestrained mice with 48 h of reperfusion using the excretion kinetics of an intravenous bolus of FITC-sinistrin (6 mg/100 g body weight; dissolved in sodium chloride (NaCl) 0.9% Fresenius Kabi, GmgH Estermannstraße 17 A-4020 Linz, Austria) using a miniaturized fluorometric detector (NIC-Kidney excitation 480 nm/emission 521 nm; Mannheim Pharma & Diagnostics; MediBeacon St. Louis, MO, USA). GFR was calculated using the half-life derived from the rate constant of the single exponential phase of the FITC-sinistrin excretion curve as previously described [41].

### 2.6. Real-Time PCR

We studied the cortex and the medulla separately to understand the effect of AG in both kidney regions. Total RNA was isolated using an RNeasy Mini Kit (#74014, Qiagen, Germantown, MD, USA) according to the manufacturer’s directions. Extracted RNA was quantified at 260 nm in a NanoDrop 2000 Spectrophotometer (NanoDrop Technologies, Madison, WI, USA), and the integrity of the RNA was assessed by agarose gel electrophoresis. cDNA was prepared from total RNA (1 μg) using a reverse transcription system (random hexamers (C-1118), Improm II Reverse Transcriptase System (A-3802) from Promega). Then, PCR was duplicated for each experiment (HotStartTaq DNA polymerase from Qiagen or BRILLIANT III ULTRA-FAST SYBR GREEN QPCR (Stratagene). Amplicons were detected for real-time fluorescence detection (Rotor-Gene Q, Qiagen, Germantown, MD, USA). The primers used are detailed in Table S1. Relative mRNA abundance was calculated using Ct values and normalized to the relative abundance of each transcript.

### 2.7. Western Blot Assay

We studied the cortex and the medulla separately to understand the effect of AG in both kidney regions. Western blot was realized as was previously published with some modifications [18]. Briefly, the renal cortex and the medulla were dissected and homogenized with an Ultra-Turrax homogenizer (Model PRO-200, PRO Scientific, Vernon Hills, IL, USA) in ice-cooled 10 mM Tris·HCl buffer at pH 7.4, supplemented with 1 mM EDTA, 1 mM EGTA, 0.25 M sucrose, 1% vol/vol Triton X-100, and a protease inhibitor cocktail (Complete Mini, Roche Applied Science # 5892970001). Tissue homogenates were subject to centrifugation. Step one was 900× *g* by 10 min. Next, the tissue was sonicated for 30 s on ice, vortexed, and centrifuged again by 2400× *g* and 17,000× *g* by 5 min. All procedures were held at 4 °C. Total proteins in supernatants were measured using the BCA Protein Assay Kit (ThermoFisher Scientific, Rockford, IL, USA), and samples were stored at −80 °C. The antibodies were: anti-HSP27 (sc-13132; Santa Cruz, OR, USA) and mouse anti-β-actin (A5441; Sigma, St. Louis, MO, USA) antibodies. Secondary antibodies were anti-mouse (A-21257) or anti-rabbit (A-21039) IgG conjugated with Alexa Fluor-750 (Thermo Scientific, South San Francisco, CA, USA). The resulting band intensities were quantified using Odyssey equipment and Image Studio Lite software (version 5.25; Li-Cor, Lincoln, NE, USA).

### 2.8. Histochemical Analysis and Tissue Damage Determination

Kidneys were fixed in 10% formalin included in paraffin. The kidneys were fixed in 10% buffered formalin, embedded in paraffin, sectioned, dewaxed, rehydrated, rinsed in water, and stained with hematoxylin-eosin (HE). The morphologic analysis was carried out in a blinded manner as detailed previously by a physician/pathologist [18]. The cortex and the medulla were evaluated for epithelial necrosis, loss of brush border, tubular dilation, and tubular congestion, among other kidney alterations observed in AKI response.

### 2.9. Statistical Analysis

Differences between groups were analyzed using one-way ANOVA, nonparametric test, and Kruskal–Wallis test using GraphPad Prism Software. The levels of significance were represented by * *p* < 0.05 or ** *p* < 0.005. Data were the mean ± standard error of the mean (SEM).

## 3. Results

### 3.1. AG Prevented Kidney Injury due to Renal Ischemia and Reperfusion (IR)

**Figure 1:**
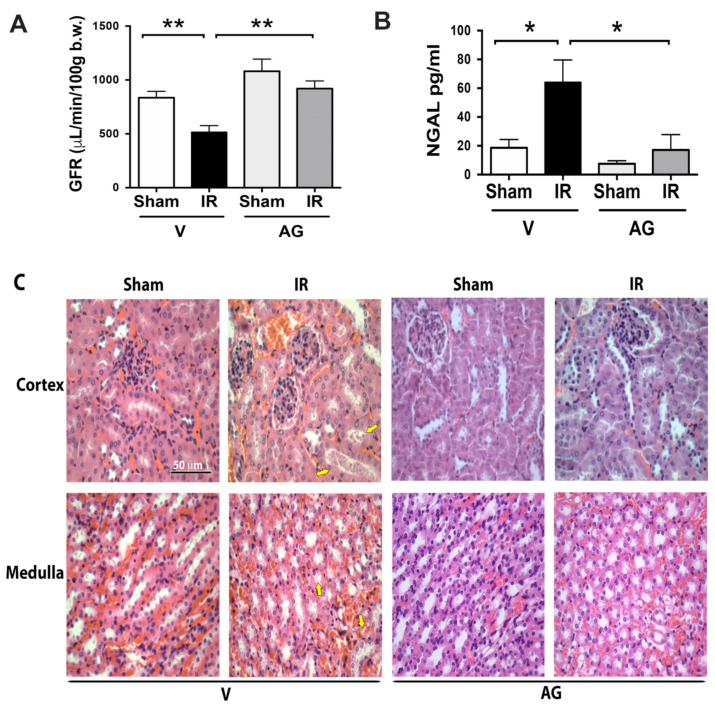
AG prevented the kidney damage induced by renal IRI. BALB/c mice were treated with either vehicle or AG (50 mg/kg i.p) before sham or ischemia-reperfusion (IR) surgery and subjected to 30 min of ischemia and 48 h of reperfusion. (**A**) GFR (µL/min/100 g body weight) was determined in sham (n = 5), IR (n = 6), AG-sham (n = 5), and AG-IR (n = 6). (**B**) Neutrophil gelatinase-associated lipocalin (NGAL) was measured by ELISA in blood samples in sham (n = 9), IR (n = 6), AG-sham (n = 5), and AG-IR (n = 6). The bar graphs represent mean ± SEM, and the data were analyzed using ANOVA and non-parametric Kruskal–Wallis test. * *p* < 0.05 and ** *p* < 0.005. (**C**) Histological analysis: representative hematoxylin/eosin (H/E) staining of the cortex and the medulla kidney sections. Yellow arrows indicate areas with evident kidney acute tubular necrosis (ATN) characterized by loss of nucleus in tubules or intratubular cellular detritus. Three kidneys for each protocol were analyzed. Representative images correspond to 400X scale bar = 50 µm.

Kidneys from Balb/c adult mice were subjected to 30 min ischemia and 48 h of reperfusion as described previously [18]. We studied kidney damage by measuring glomerular filtration rate (GFR), acute kidney injury biomarker (neutrophil gelatinase-associated lipocalin, NGAL), and histological analysis. The GFR decreased by approximately 50% in mice subjected to renal IR compared with sham (476.7 ± 56.0 and 869.2 ± 62.0 µL/min/100 g body for IR and sham, respectively; *p* < 0.05). In addition, the blood NGAL concentration was significantly higher (3.5-fold) from mice subjected to the IR compared with sham mice (64.1 ± 37.1 and 18.5 ± 5.7 pg/mL for IR and sham, respectively; *p* < 0.05). Interestingly, the AG administration (50 mg/kg) 30 min before IR protocol prevented the GFR downregulation (918.4 ± 79.0 µL/min/100 g body weight; *p* < 0.05) and avoided the NGAL upregulation (16.8 ± 16.8 pg/mL; *p* < 0.05) induced by IR (Figure 1). The kidney morphology was analyzed earlier (at 30 min of reperfusion) than 48 h of reperfusion. The data revealed normal kidney morphology in the cortex and the medulla of the control animals (sham group). Glomeruli and tubules had normal structures (Figure 1C). In contrast, acute tubular necrosis (ATN) was present in the kidney in the IR group, characterized by loss of the brush border, intratubular cellular detritus, loss of the nucleus, and vascular congestion (Figure 1C, arrows). In contrast, no signal of ATN was observed in the kidneys from sham and IR mice treated with AG. Altogether, these results suggested the renoprotective effect of AG against ischemia and reperfusion injury.

**Figure 2:**
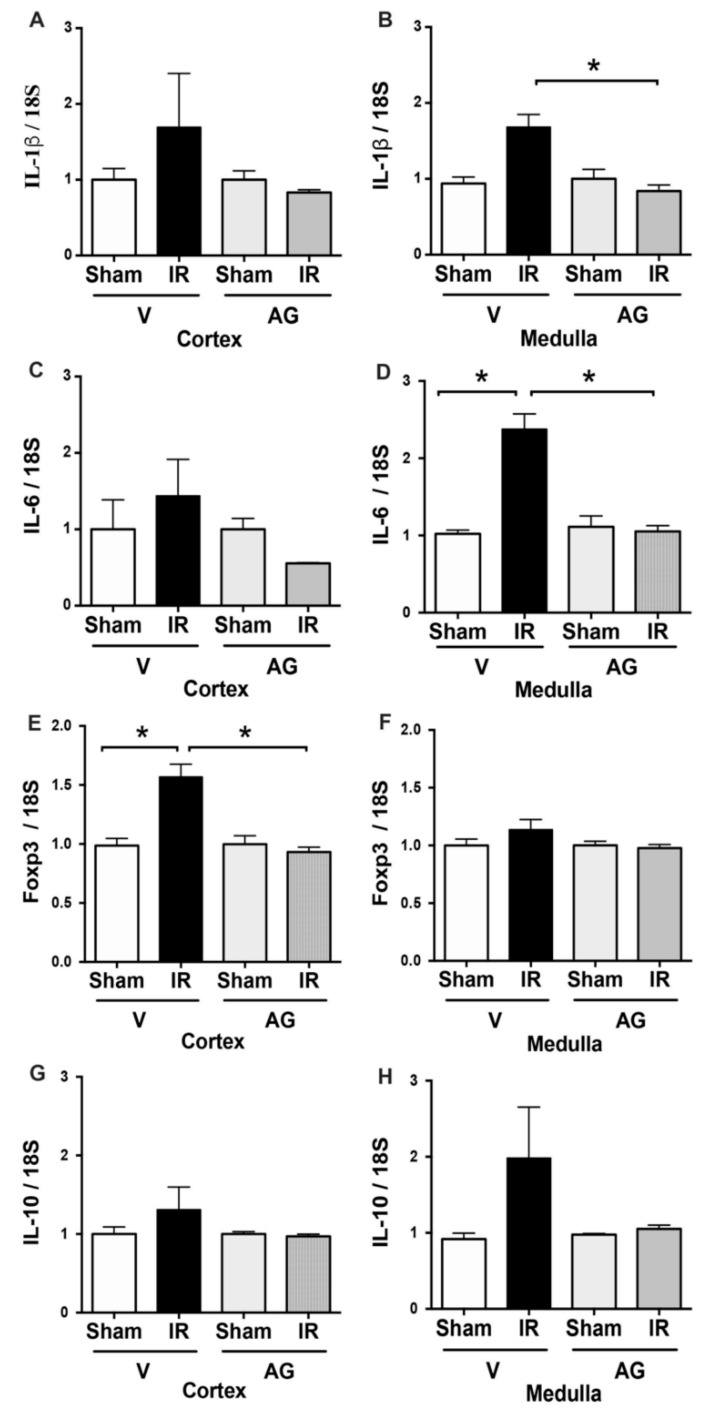
AG inhibited the IL-1β, IL-6, Foxp3, and IL-10 mRNA upregulation observed during IR. BALB/c mice were treated with either vehicle or AG (50 mg/kg i.p) before sham or subjected to 30 min of ischemia and 48 h of reperfusion (IR). Cytokine expression was determined by qPCR in sham (n = 5), IR (n = 5), AG-sham (n = 6), and AG-IR (n = 6). (**A**) IL-1β mRNA in the cortex. (**B**) IL-1β mRNA in the medulla. (**C**) IL-6 mRNA in the cortex. (**D**) IL-6 mRNA in the medulla. (**E**) Foxp3 mRNA in the cortex. (**F**) Foxp3 mRNA in the medulla. (**G**) IL-10 mRNA in the cortex. (**H**) IL-10 mRNA in the medulla. The bar graphs represent mean ± SEM, and the data were analyzed using ANOVA and non-parametric Kruskal–Wallis test. * *p* < 0.05.

In addition, inflammation is one of the most critical stages in renal injury after ischemia. We investigated the mRNA expression of two pro-inflammatory cytokines (IL-1β and IL-6) and two regulatory factors of inflammation (IL-10 and Foxp3). We did not find a significant upregulation of IL-1β by IRI in the cortex or the medulla. However, we observed a significant upregulation of IL-6 mRNA in the renal medulla (but not in the cortex) by IR compared to the sham group. Besides, renal IR upregulated the Foxp3 mRNA expression in the kidney cortex but not in the medulla (Figure 2C,D). In addition, the IL-10 mRNA was not significantly upregulated by IR in the cortex or the medulla (Figure 2). Interestingly, AG reduced IL-1β and IL-6 mRNA levels in the kidney medulla sections, preventing the Foxp3 upregulation in the cortex. These data suggest that AG prevents the upregulation of inflammatory elements observed during renal IRI.

**Figure 3:**
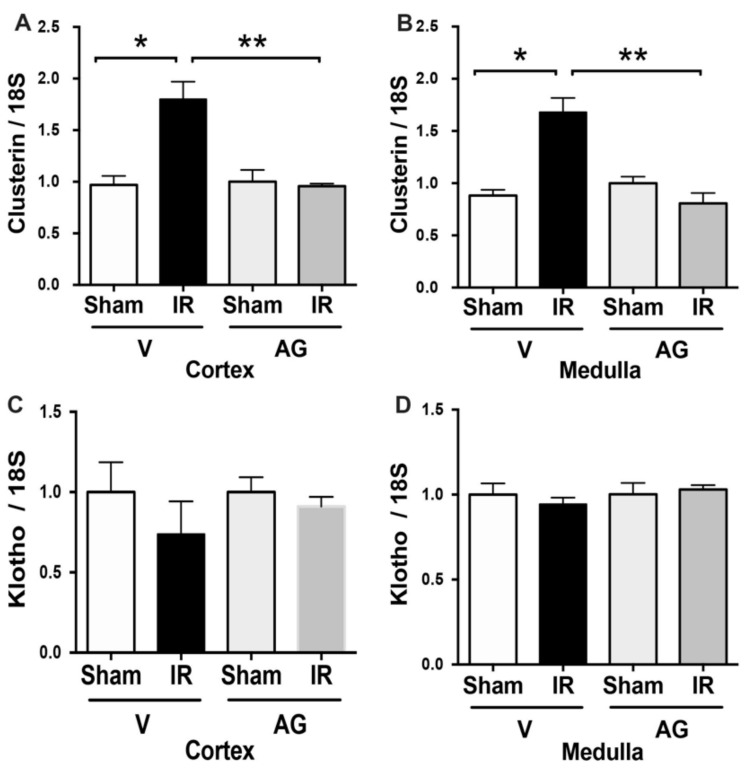
AG inhibited the upregulation of clusterin mRNA observed during IR, but not for klotho. BALB/c mice were treated with either vehicle or AG (50 mg/kg i.p) before sham or subjected to 30 min of ischemia and 48 h of reperfusion (IR). The clusterin and the klotho mRNA expressions were determined by qPCR in sham (n = 5), IR (n = 5), AG-sham (n = 6), and AG-IR (n = 6). (**A**) Clusterin mRNA in the cortex. (**B**) Clusterin mRNA in the medulla. (**C**) Klotho mRNA in the cortex (**D**) Klotho mRNA in the medulla. The bar graphs represent mean ± SEM, and the data were analyzed using ANOVA and non-parametric Kruskal–Wallis test. * *p* < 0.05, ** *p* < 0.005.

Besides, clusterin (CLU) and klotho are two markers of kidney protection by renal IR. We previously reported that CLU was increased in kidneys subjected to IRI, and it was downregulated by l-NIL (a specific iNOS inhibitor) [18]. Here, we also showed that mRNA levels of CLU were significantly increased in both cortex and medulla in the IRI animals compared with sham. Remarkably, the AG administration decreased the upregulation of CLU observed by IRI (Figure 3A,B). On another side, we studied the expression of klotho (a renoprotective and anti-aging gene highly expressed in the kidney), and we did not observe changes in the mRNA klotho expression in either cortex or medulla kidney sections by IRI or AG.

**Figure 4:**
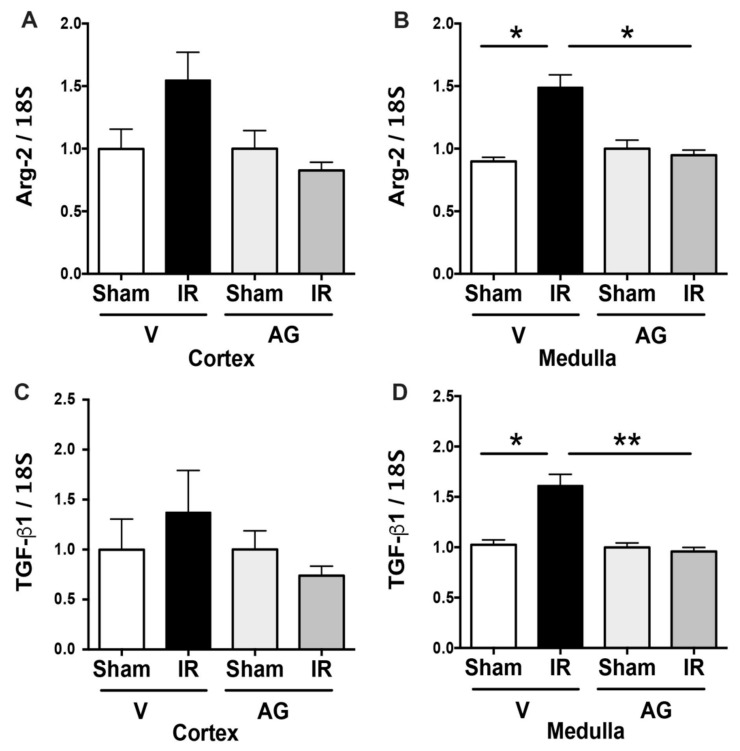
AG inhibited the upregulation of Arg-2 and TGF-β1 mRNA observed during IR. BALB/c mice were treated with either vehicle or AG (50 mg/kg i.p) before sham or subjected to 30 min of ischemia and 48 h of reperfusion (IR). The Arg-2 and the TGF-β1 mRNA expressions were determined by qPCR in sham (n = 5), IR (n = 5), AG-sham (n = 6), and AG-IR (n = 6). (**A**) Arg-2 mRNA in the cortex. (**B**) Arg-2 mRNA in the medulla. (**C**) TGF-β1 mRNA in the cortex (**D**) TGF-β1 mRNA in the medulla. The bar graphs represent mean ± SEM, and the data were analyzed using ANOVA and Kruskal–Wallis test. * *p* < 0.05, ** *p* < 0.005.

Finally, we showed that Arg-2 and TGF-β1 mRNA levels were significantly increased in the medulla in the IRI animals compared with sham. In the cortex, we observed similar results, but it was not statistically significant. Remarkably, the AG administration decreased the upregulation of Arg-2 and TGF-β1 observed by IRI (Figure 4).

### 3.2. AG Prevented Oxidative Stress

**Figure 5:**
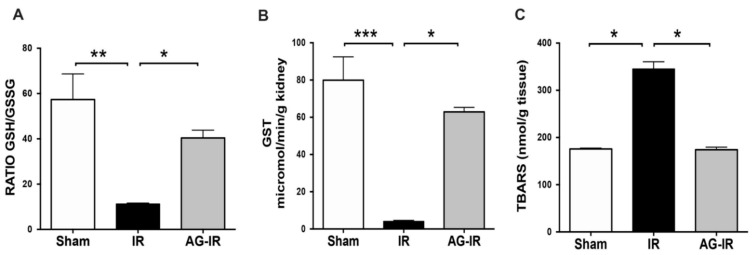
AG ameliorated the oxidative stress induced by renal IR. BALB/c mice were treated with either vehicle or AG (50 mg/kg i.p) before sham or IR surgery and subjected to 30 min of ischemia and 48 h of reperfusion. GSH:GSSG ratio, GST activity, and TBARS (lipoperoxidation) were determined in whole kidneys in sham (n = 5), IR (n = 5), and AG-IR (n = 6) (**A**) GSH:GSSG ratio. (**B**) Glutathione S-transferase (GST) activity. (**C**) Thiobarbituric acid reactive substances (TBARS) levels. Bar graph represents mean ± SEM, and data were analyzed by ANOVA non-parametric Kruskal–Wallis analysis, * *p* < 0.05, ** *p* < 0.005, *** *p* < 0.0005.

We selected a panel of oxidative stress markers according to our previous publication [18]. We studied the oxidative stress in the whole kidney, assaying the GSH-to-GSSG ratio as a measure of the soluble antioxidant status (Figure 5A), the glutathione S-transferase (GST) activity as an antioxidant mechanism (Figure 5B), and the TBARS as a readout for lipid peroxidation (Figure 5C). Mice exposed to renal IR had 3.8-fold reduced levels of GSH:GSSG ratio compared to the sham group (15.5 ± 4 and 57.3 ± 1 for IR and sham, respectively; *p* < 0.05. Figure 5A). Besides, the GST activity was deeply downregulated by renal IR (4.0 ± 0.5 and 79.9 ± 12.5 µmol/min/g tissue for IR and sham, respectively; *p* < 0.05. Figure 5B). In consequence, the renal lipid peroxidation increased by renal IR (345.3 ± 15 and 175.8 ± 1.7 nmol/g-tissue for IR and sham, respectively; *p* < 0.05. Figure 5C). Noteworthy, AG treatment before renal IR increased the GSH:GSSG (33.4 ± 3.5 for AG-IR) ratio, recovered the GST activity (63.1 ± 3.0 µmol/min/g tissue for AG-IR), and reduced the oxidative damage (TBARS: 180.3 ± 3.8 nmol/g tissue for AG-IR) levels. All these data suggested that AG prevented oxidative stress in kidneys exposed to IRI.

### 3.3. Molecular Sensors of AG Antioxidant Effect

**Figure 6:**
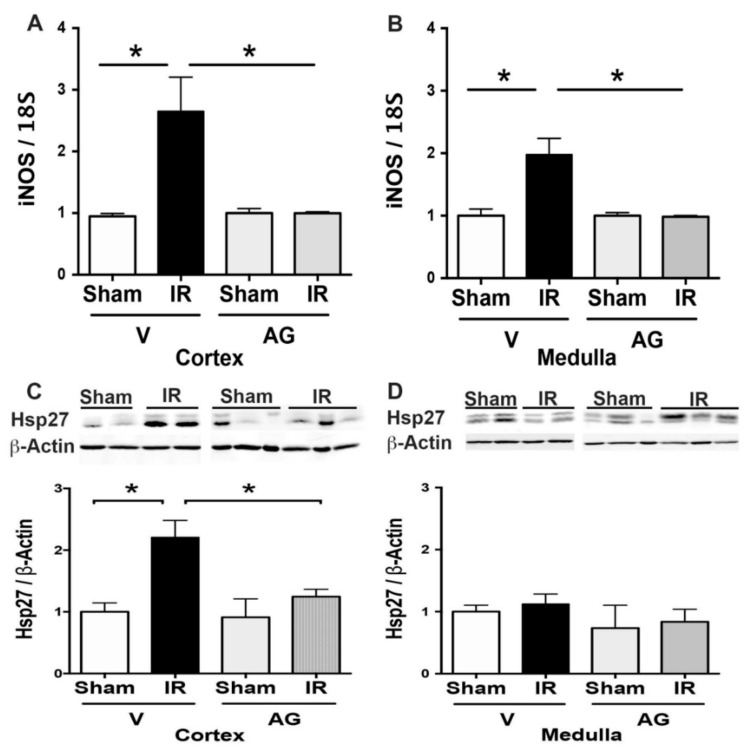
AG inhibited upregulation of iNOS and Hsp27 observed during IR. BALB/c mice were treated with AG (50 mg/kg i.p.) before sham or subjected to 30 min of ischemia and 48 h of reperfusion (IR). The iNOS mRNA expression was determined by qPCR and Hsp27 protein by Western blot in sham (n = 5), IR (n = 5), AG-sham (n = 6), and AG-IR (n = 6). (**A**) INOS mRNA in the cortex. (**B**) INOS mRNA in the medulla. (**C**) Hsp27 protein in the cortex. (**D**) Hsp27 protein in the medulla. The bar graph represents mean ± SEM, and the data were analyzed by using ANOVA and non-parametric Kruskal–Wallis test. * *p* < 0.05.

During renal IRI, the iNOS activity induces oxidative stress [42,43,44], and Hsp27 prevents it [45,46]. Here, we observed an upregulation of iNOS mRNA by renal IR in the cortex and the medulla (Figure 6A,B). Noteworthy, AG significantly prevented the upregulation of iNOS by renal IR. On the other hand, Hsp27 protein expression was significantly upregulated by IR in the cortex kidney, and AG avoided its expression. These data suggested that the antioxidant effect of AG prevented the upregulation of iNOS and Hsp27 (Figure 6C,D) involved in the oxidant response in the kidney by IRI.

### 3.4. Effect of AG on Endothelial Markers Expression

**Figure 7:**
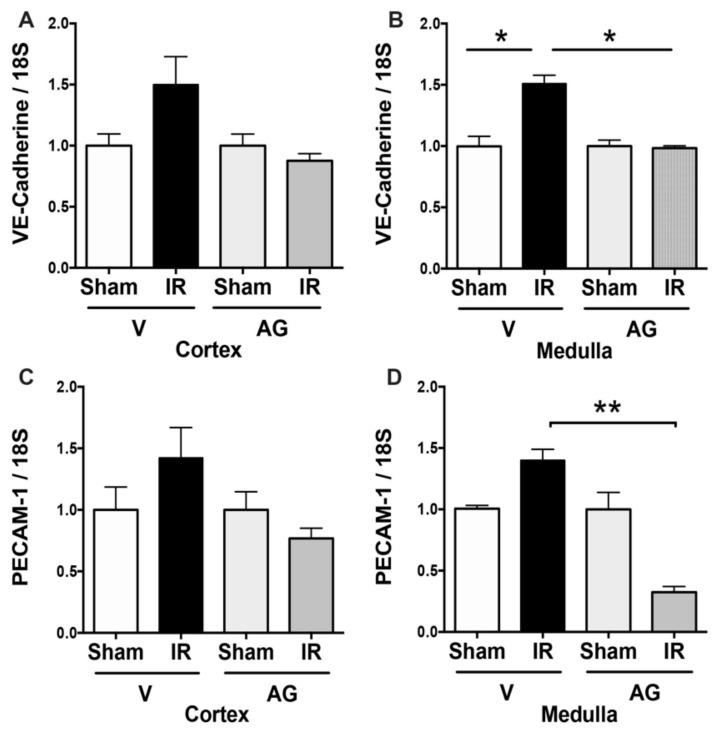
AG prevented VE-cadherin and PECAM upregulation induced by IR. BALB/c mice were treated with either vehicle or AG (50 mg/kg i.p) before sham or subjected to 30 min of ischemia and 48 h of reperfusion. VE-cadherin and PECAM-1 expressions were determined by qPCR in sham (n = 5), IR (n = 5), AG-sham (n = 6), and AG-IR (n = 6). (**A**) VE-cadherin mRNA in the cortex. (**B**) VE-cadherin mRNA in the medulla. (**C**) PECAM-1 mRNA in the cortex. (**D**) PECAM-1 mRNA in the medulla. The bar graph represents mean ± SEM using ANOVA non-parametric Kruskal–Wallis test. * *p* < 0.05, ** *p* < 0.005.

To examine the mechanisms involved in the protection induced by AG treatment against IRI, we studied the mRNA expression of endothelial markers VE-cadherin and PECAM-1 (platelet/endothelial cell adhesion molecule 1 or CD31) [32]. The results demonstrated that VE-cadherin mRNAs were significantly upregulated in the renal medulla in mice subjected to 48 h of reperfusion. The PECAM mRNA also increased, but it was not statistically significant. The administration of AG before IR significantly reduced VE-cadherin and PECAM mRNA (Figure 7). The findings suggested that AG prevented the endothelial dysfunction induced by IRI, most likely through its antioxidant effects.

### 3.5. Effect of AG on Mesenchymal Markers Expression: Vimentin, Fascin, and Hsp47

**Figure 8:**
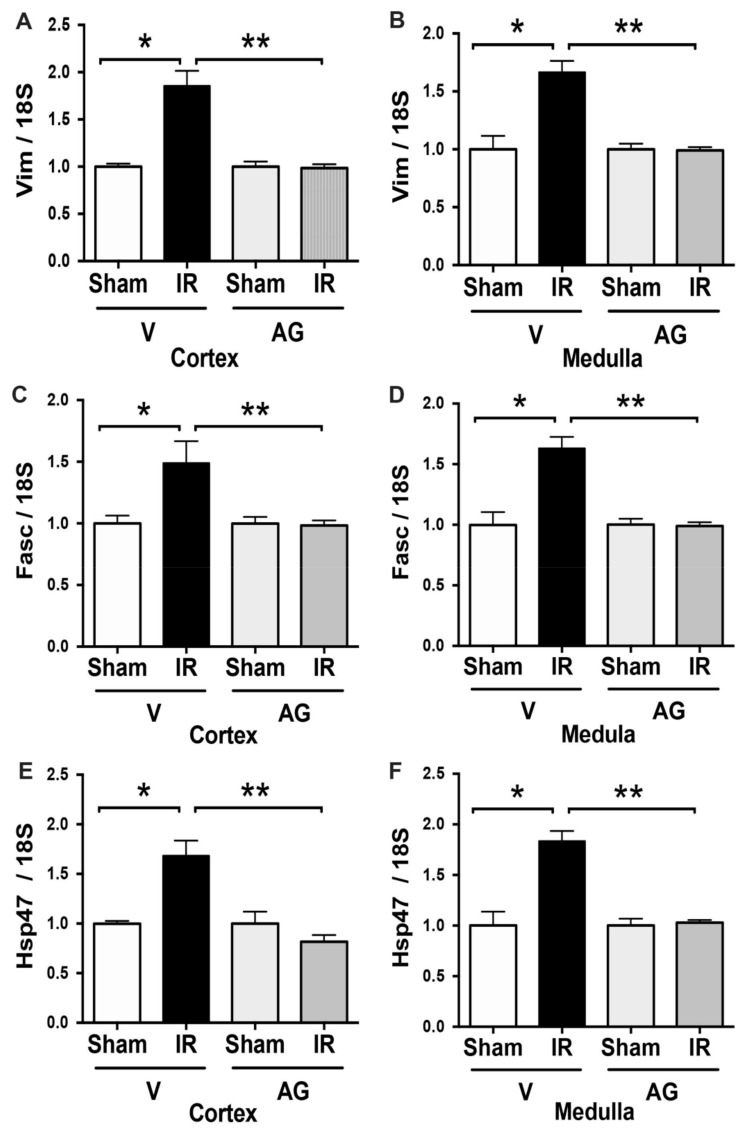
AG inhibited vimentin, fascin, and Hsp47 mRNA upregulation during IR. BALB/c mice were treated with either vehicle or AG (50 mg/kg i.p) before sham or subjected to 30 min of ischemia and 48 h of reperfusion (IR). Vimentin, fascin, and Hsp47 mRNA expression were determined by qPCR in sham (n = 5), IR (n = 5), AG-sham (n = 6), and AG-IR (n = 6). (**A**) Vimentin mRNA in the cortex. (**B**) Vimentin mRNA in the medulla (**C**) Fascin mRNA in the cortex. (**D**) Fascin mRNA in the medulla. (**E**) Hsp47 mRNA in the cortex. (**F**) Hsp47 mRNA in the medulla. The bar graph represents mean ± SEM, and the data were analyzed using ANOVA non-parametric Kruskal–Wallis test. * *p* < 0.05, ** *p* < 0.005.

Vimentin is an intermediate filament used as a marker for the state of differentiation and is expressed in kidney mesenchymal cells but not in tubular epithelial cells. On the other hand, fascin-1 is an actin-bundling protein involved in cell migration and expressed in the mesenchymal phenotype. The Hsp47 is a pro-fibrotic protein required for procollagen biosynthesis and is also suggested to be increased by mesenchymal phenotype. The mRNA expressions of vimentin, fascin, and Hsp47 were upregulated in the cortex and the medulla kidney in mice subjected to IR (Figure 8). Notably, AG treatment significantly prevented vimentin, fascin, and Hsp47 upregulation observed by IR in both kidney sections. These data suggested that the antioxidant effect of aminoguanidine prevented the expression of mesenchymal transition markers in the kidney induced by IRI.

## 4. Discussion

We found an antioxidant effect of AG in the murine model of ischemic (30 min) and reperfusion (48 h) injury. The major findings of this study are: (1) the AG pretreatment recovered GSH levels and GST activity in the kidney during renal IRI, therefore reducing the lipoperoxidation levels in the whole kidney. (2) The upregulation of iNOS (cortex and medulla) and Hsp27 (cortex) by IRI was prevented by AG pretreatment. (3) The upregulation of clusterin, Arg-2, and TGF-β1 by IRI was also prevented by AG. (4) The kidney damage biomarkers (GFR, NGAL, and histology) observed by IRI were prevented by AG. (5) AG inhibited IL-1β, IL-6, and Foxp3 mRNA upregulation induced by IRI. (6) The AG prevented the endothelial dysfunction (VE-cadherin and PECAM) and the upregulation of mesenchymal transition markers (vimentin, fascin1, and Hsp47) observed by IRI.

### 4.1. Antioxidant Effect of AG and Prosurvival Genes

In the kidney, after injury, NADPH oxidase, mitochondria, and inducible nitric oxide synthase are the primary sources of oxidative stress [14,15]. The glutathione S-transferase (GST) is an isozyme whose primary function is to detoxify and neutralize a wide variety of electrophilic molecules by mediating their conjugation with reduced glutathione [47]. Low levels of GST increase the presence of oxidative stress and reduce the GSH:GSSG ratio, therefore increasing levels of lipoperoxidation. We previously described the downregulation of GST activity by renal IRI in mice [18]. Our results indicated that the administration of AG before renal IR increased the levels of the GSH, recovered the GST activity, and reduced oxidative damage. To our knowledge, this is the first report showing that, during renal IRI in mice, AG recovered GSH levels and GST activity, reducing the lipoperoxidation.

A high level of oxidative stress can lead to the induction of different pro-survival signals. Here, we showed increased oxidative stress and upregulation of Hsp27 only in the cortex during IRI. During oxidative stress, Hsp27 functions as an antioxidant in cells, lowering ROS levels by reducing intracellular iron levels and raising intracellular levels of reduced glutathione [48]. Hsp27 confers cytoprotection from ischemia [49] by stabilizing the actin cytoskeleton, displaying an anti-apoptotic function, reducing ATP depletion, and inhibiting the reactive oxygen species [50]. The antioxidant effect of Hsp27 was described in the heart and the brain [51], and, together with the A1 adenosine receptor (AR), it works as a renal protector against IRI [52]. Therefore, the present data showed that AG inhibited the upregulation of Hsp27 during renal IRI, which could be a consequence of the antioxidant effect of this compound.

Clusterin (CLU) is an extremely sensitive biosensor to exogenous or endogenous stress, particularly free radicals and their derivatives. Several studies demonstrated that CLU has a cytoprotective role against the deleterious effects of oxidants, working as an antioxidant protein [53]. Recently, it was observed that glomerular CLU is upregulated in diabetic nephropathy and may have a protective effect against oxidative stress-induced apoptosis in podocytes [54]. Induction of CLU during IRI was proposed as a survival function in response to high levels of oxidative stress through a mechanism involved in survival autophagy [55]. In addition, autophagy protects renal tubular cells against ischemia [56]. The prevention of Hsp47, Arg-2, and TGF-β1 mRNA levels upregulation by the AG treatment suggested inhibition of the kidney injury process associated with the development of fibrosis. The present work provided the first evidence that AG inhibits CLU, Arg-2, TGF-β1, and Hsp27 upregulation by IRI (Figure 3, Figure 4 and Figure 6, respectively), suggesting that the antioxidant effect of AG alleviates the injury induced by IR.

### 4.2. Antioxidant Effect of AG and Endothelial Injury

During renal IRI, endothelial damage is observed. Previously, it was described that, in a murine model of IRI, the renal VE-cadherin mRNA levels were downregulated in the first hour of reperfusion. However, after 24 h of reperfusion, the VE-cadherin mRNA levels were significantly upregulated compared to the sham group as a compensatory effect [57]. We found that VE-cadherin and PECAM-1 mRNA were upregulated after 48 h of reperfusion compared with the sham group. Remarkably, the antioxidant effect of AG prevented this phenomenon. Interestingly, PECAM could result in the transmission of a pro-survival signal suppressing the mitochondria-dependent Bax-mediated intrinsic apoptotic pathway or inhibiting cytochrome c release from mitochondria [58]. These data suggested that the antioxidant effect of AG had a protective effect on renal endothelium during IRI.

Additionally, we noted upregulation of vimentin, fascin, and Hsp47 after IRI. Previously, Xu-Dubois et al. demonstrated increased expression of these markers in early posttransplant biopsies, associated with poor renal graft recovery and/or late graft dysfunction and endothelium injury [36]. Therefore, our data suggested that AG could have a renoprotective effect during kidney transplants, reducing endothelium dysfunction. Future studies are needed to determine the role of AG during kidney transplantation.

### 4.3. AG and Oxidative Stress in the Kidney

The pathophysiology of renal IR is a complex process regulated by several intracellular pathways, in which reactive oxygen and nitrogen species (ROS/RNS) appear to be central mediators of detrimental effects. During the renal IR, the reperfusion phase results in increased ROS production [12,15]. After the injury, the primary source of oxidative stress in the kidney comes from NADPH oxidase, mitochondria, and inducible nitric oxide synthase [14,15]. Overproduction of inflammatory mediators and ROS can lead to perturbations of microcirculation and tissue oxygenation [59]. On another note, NO produced by iNOS enhances oxidative stress and impairs renal function [60]. The NO combines with the superoxide radical and forms the cytotoxic metabolite, peroxynitrite, which causes cell membrane damage through protein nitration [61]. Thus, an increase in nitrotyrosine levels in patients with acute kidney injury is associated with overall mortality [62]. Therefore, ROS and NO can inhibit the compensatory changes in renal hemodynamics produced by IR after renal ischemia, therefore decreasing tubular function [63,64]. Our animal model of renal IRI produced elevated oxidative stress (inactivation of GST, increased GSSG levels, and elevated lipid peroxidation), decreased the glomerular filtration rate, and increased kidney damage (NGAL and clusterin). Noteworthy, the pretreatment of AG 30 min before IRI reduced the oxidative stress, and the renoprotective effect was observed.

Besides ischemia, other pathophysiological mechanisms can contribute to kidney damage, such as antibiotics aminoglycosides and chemotherapeutic agents. Data on the role of AG in kidney toxicity have been limited to studies with rats. In a cyclophosphamide (CP) induced nephrotoxicity model, AG protects the kidney from oxidative stress. The CP administration (50 mg/kg body weight, i.p.) induced oxidative stress in the kidney by increase of lipoperoxidation, protein oxidation, depletion of GSH, as well as loss of catalase, glutathione peroxidase (GPx), myeloperoxidase (MPO), and GST. The pretreatment with AG (200 mg/kg body weight, i.p. 1 h before CP administration) prevented CP-induced oxidative stress, decreased lipoperoxidation, protein oxidation, GSH levels, and recovered GPx catalase, GST, and MPO activities [65]. Another study explored the protective effect of AG on nephrotoxicity induced by gentamicine (GEN). The GEN administration to control group rats increased renal MDA and NO levels but decreased GPx, SOD, CAT activities, and GSH content. The AG administration with GEN injection significantly decreased MDA and NO generation and increased GPx, SOD, CAT activities, and GSH abundance [66]. Using a mice model of IR, our finding reproduced the antioxidant effect of AG observed by cyclophosphamide and gentamicine oxidative stress in rats. In our case, we used a lower dose of AG (50 mg/kg body weight, i.p), and we found that the antioxidant effect of AG (recovered GSH, reduced lipoperoxidation, and increased GST activity) was associated with inhibition of iNOS, Hsp27, endothelial activation (VE-cadherin and PECAM) and mesenchymal markers (vimentin, fascin1, and Hsp47). Besides, we studied the mRNA of two pro-inflammatory (IL-1β and IL-6) and two anti-inflammatories (Foxp3 and IL-10) elements in the cortex and the medulla. After 48 h of reperfusion, we found significant upregulation of Foxp3 mRNA in the cortex. On the other hand, IL-1b and IL-6 were upregulated by IR (48 h reperfusion) in the medulla but not the anti-inflammatory elements (Foxp3 and IL-10). Remarkably, the pro-inflammation–anti-inflammatory equilibrium was entirely abolished by AG. Finally, AG improved the glomerular filtration rate and reduced the tubular epithelial damage (NGAL, clusterin, arg-2, and TFG-β1). Therefore, the antioxidant properties of AG prevented acute kidney injury markers and improved kidney function. Consequently, AG is a promising pharmacological compound to treat the deleterious effect of acute renal ischemia and reperfusion injury.

## 5. Conclusions

The present study demonstrated the beneficial effect of AG on renal IRI through the suppression of oxidative stress, iNOS, Hsp27, Arg-2, and TFG-β1. In addition, AG prevented endothelium activation mesenchymal transition markers and inflammation elements. Moreover, AG reduced kidney injury and improved kidney function. These results may be of potential clinical relevance, and the protective effect of AG as a therapeutic strategy may be of value in the future.

## Data Availability

Data is contained within the article and supplementary materials.

## Supplementary Materials

The following are available online at https://www.mdpi.com/article/10.3390/antiox10111724/s1. Table S1. Primers for Real-Time quantitative PCR.
