# Supplementary material for: Aminoguanidine Prevents the Oxidative Stress, Inhibiting Elements of Inflammation, Endothelial Activation, Mesenchymal Markers, and Confers a Renoprotective Effect in Renal Ischemia and Reperfusion Injury
Source: Antioxidants (Basel). 2021 Oct 28;10(11):1724. doi: 10.3390/antiox10111724 (PMC8614713; doi:10.3390/antiox10111724)
Supplement: Supplementary file 1 [file antioxidants-10-01724-s001.zip › antioxidants-1384035-supplementary.pdf]

Gen	Sequence	Type	TM (°C)	Specie	Amplicon size (bp)
IL-1*β*	5´-CAAATCTCGCAGCAGCACA-3´	Forward	56.2	Mouse	121
	5´-TCATGTCCTCATCCTGGAAGG-3´	Reverse	56.2	Mouse	121
IL-6	5´-TACCATAGCTACCTGGAGTAC-3´	Forward	52.6	Mouse	150
	5´-AATTGGGGTAGGAAGGACTAT-3´	Reverse	52.7	Mouse	
IL-10	5´-GGT TGC CAA GCC TTA TCG GA-3´	Forward	57.7	Mouse	191
	5´-ACC TGC TCC ACT GCC TTG CT-3´	Reverse	61.9	Mouse	
FOXP3	5´-AACCTGAGCCTGCACAAGTGCTTT-3´	Forward	61.6	Mouse	134
	5´-TGAGGTCAAGGGCAGGGATTGGA-3´	Reverse	62.4	Mouse	
Hsp47	5´-TGCAGAAACATCTGGCAGGAC-3´	Forward	57.8	Mouse	167
	5´-CCCGTAGATGTCTTGGTCAAAGG-3´	Reverse	57.4	Mouse	
Fascin 1	5'-AAGCTGATTAACCGCCCCAT-3'	Forward	57.0	Mouse	157
	5'-TGCCCGTGGAGTCTTTGATG-3'	Reverse	57.6	Mouse	
Vimentin	5'-GTGGATCAGCTCACCAACGA-3'	Forward	57.2	Mouse	160
	5'-AAGCATTGTCAACATCCTGTCTG-3'	Reverse	55.6	Mouse	
Clusterin	5'-TTGACTCTGACCCCATCACA-3'	Forward	55.7	Mouse	110
	5'-GCTTTTCCTGCGGTATTCCT-3'	Reverse	55.4	Mouse	
PECAM 1	5'-TCCAAAAAGACAAGGCGATTGT-3'	Forward	55.3	Mouse	164
	5'-GGGAAACAGCTCTGTTATGTTGA-3'	Reverse	55.2	Mouse	
VE-Cadherin 5	5'-CAGCATCATGCAGGGCGAGTA-3'	Forward	59.8	Mouse	145
	5'-AGTGGGGTCTGTGGCCTCAAT-3'	Reverse	61.0	Mouse	
TGF-*β*1	5'-CGAAGCGGACTACTATGCTAA-3'	Forward	54	Mouse	130
	5'-TTCCCGAATGTCTGACGTATT-3'	Reverse	53.8	Mouse	
Arginase-2	5'-CAGGTGGCTACAGCTGTGTCAC-3´	Forward	60.1	Mouse	118
	5'-TCCGCATGAGCATCAACCCAG-3'	Reverse	60.0	Mouse	
Klotho	5'-TATTGATGGCGACTACCC-3'	Forward	51.2	Mouse	167
	5'-GGCGGAACTTCATGTTAG-3'	Reverse	50.7	Mouse	
iNOS	5'-TCCTGCCTCATGCCATTGAGTT-3'	Forward	59.0	Mouse	81
	5'-GCCTGGCCAGATGTTCCTCTATTT-3'	Reverse	59.1	Mouse	
18S	5'-CTCTAGATAACCTCGGGCCGATCG-3'	Forward	60.2	Mouse	170
	5'-GATGTGGTAGCCGTTTCTCAGGCT-3'	Reverse	60.9	Mouse	
